# Plastic Bronchitis in Children: A Review of 55 Cases over a 10-Year Period

**DOI:** 10.1155/2024/9271324

**Published:** 2024-06-25

**Authors:** Xiaowen Chen, Shangzhi Wu, Zhanhang Huang, Yuneng Lin, Jiaxing Xu, Qingyun Xu, Dehui Chen

**Affiliations:** Department of Pediatrics The First Affiliated Hospital of Guangzhou Medical University, Guangzhou, Guangdong, China

## Abstract

**Objective:**

To summarize the clinical characteristics and treatment experiences of patients with plastic bronchitis (PB).

**Methods:**

All patients who were diagnosed with PB by bronchoscopic removal of tree-like casts at a single institution from January 2012 to May 2022 were retrospectively reviewed. Demographic and clinical data were retrieved from electronic patient records.

**Results:**

A total of 55 patients, with a median age of 5.3 years, were eligible for the study. Nineteen cases had underlying diseases, among which asthma was the most common. The median course of the disease before admission was 11 days. Clinical symptoms were characterized by cough and fever, while moist rales (78.2%) and dyspnea (61.8%) were the most common signs. The most common laboratory finding was elevated C-reactive protein (58.2%). Patchy opacity was the most frequent radiographic finding (81.2%), followed by consolidation (60.0%) and pleural effusion (43.6%). Respiratory pathogens were detected in 41 cases, and *M. pneumoniae* was the most common one (41.8%), followed by adenovirus (20.0%) and influenza B virus (10.9%). The casts were removed by alveolar lavage, combined with ambroxol immersion (63.6%) and forceps (30.9%). Patients received an average of 2.3 bronchoscopies, and the median time for the first procedure was 3 days after admission. Antibiotics were given to all patients, methylprednisolone to 33 (60.0%), and gamma globulin to 25 (45.5%). A total of 53 cases were improved with an overall mortality rate of 3.6%.

**Conclusions:**

PB in children is characterized by airway obstruction, mostly caused by respiratory infections, and timely removal of the cast by bronchoscopy is the most effective treatment.

## 1. Introduction

Plastic bronchitis (PB) is a rare but life-threatening respiratory disease characterized by bronchial tree-like endogenous foreign bodies that partially or completely obstruct the airways [1, 2]. Due to the rapid onset and nonspecific clinical manifestations, it is easy to cause delayed diagnosis. The incidence of PB remains uncertain, with a study suggesting the prevalence of PB in children was approximately 6.8/100,000 [3]. Although PB is more common in children, many pediatricians still poorly know about it because of its low incidence and the lack of guidelines or expert consensus on the disease. The present study reviewed and summarized the clinical presentation of 55 children with PB diagnosed and treated with bronchoscopy over the past 10 years to further bridge the gaps in our understanding of the disease.

## 2. Materials and Methods

### 2.1. Patients and Data Collection

This is a retrospective study of a total of 55 pediatric patients with PB from the First Affiliated Hospital of Guangzhou Medical University over a period of 10 years from January 2012 to May 2022. There are no definitive diagnostic criteria or tests for PB. Its diagnosis is mainly clinical and is based on clinical presentation, bronchoscopic, and imaging findings [1]. PB should be suspected once the patient has the following clinical manifestations simultaneously: (1) the general symptoms including productive cough, wheezing, and dyspnea; (2) physical examination revealing decreased breath sounds at lung bases with simultaneous wheezing; (3) chest radiographs demonstrating bronchial obstruction with possible lobar atelectasis [1]. The diagnosis of PB is confirmed by a history of expectoration of branching airway casts or by removing branching casts at the time of bronchoscopy [2]. All patients who were clinically diagnosed with PB would receive bronchoscopic intervention, and the patients underwent bronchoscopy to remove the tree-like bronchial casts as the inclusion criteria in this study. In addition, patients with the following conditions were considered for glucocorticoid combination: (1) severe wheezing; (2) severe pneumonia with evident toxic symptoms, such as hypoxic toxic encephalopathy, shock, and sepsis; (3) short-term pleural effusion; (4) persistent high fever with excessive inflammatory response. For patients with severe infection, especially those with confirmed viral infection, immunoglobulin was also utilized as immune support therapy. All relevant information was retrieved from electronic patient records by investigators from the department of pediatrics. The data collected included demographic characteristics, laboratory tests, etiological examinations, treatments received, and clinical outcomes. This study was approved by the Ethics Committee of the First Affiliated Hospital of Guangzhou Medical University (reference number: 2022-K-45), and individual consent for this retrospective analysis was waived.

### 2.2. Examination and Bronchoscopy Protocol

On the first day of admission, a series of routine examinations were performed, including blood routine examination, arterial blood gas analysis, C-reactive protein (CRP), myocardial enzyme spectrum, liver and kidney function, and coagulation function tests. Before treatment with drugs especially antibiotics, nasopharyngeal swabs were taken for seven respiratory virus antigen tests, and venous blood was taken for nine etiological IgM antibodies and *Mycoplasma pneumonia* (MP) IgM antibody tests. Deep sputum was collected by aseptic aspiration or by hypertonic saline-induced cough for sputum culture. Patients received chest X-rays or CT within 72 hours before or after admission and were reexamined after treatment.

Bronchoscopy was performed after the patient fasted for 6 hours. Two milliliters of 1% lidocaine combined with 1.25 mL of Combivent was given by aerosol inhalation for surface anesthesia of the nose and pharynx 10 minutes before the operation. For patients over 8 years old who can cooperate with clinicians, lidocaine was used for local mucosal surface anesthesia in the trachea, while sufentanil combined with propofol was used for intravenous anesthesia for those under 8 years old or who cannot cooperate. Bronchoscopy with different outer diameters (Olympus, Japan) was selected according to the age of the children. During the operation, trachea, bronchus, segmental bronchus, and subsegmental bronchus were explored in turn, and alveolar lavage with normal saline via flexible bronchoscopy was performed on the lesions suggested by preoperative chest imaging. Ambroxol solution was injected locally to soften the tough casts if necessary, and even combined with forceps, cryotherapy, and other techniques to remove it. Bronchoalveolar lavage fluid (BALF) was collected for pathogen detection and culture.

### 2.3. Statistical Analysis

All data were analyzed using the statistical software package SPSS (version 21.0 for Windows; SPSS Inc.). Patients' characteristics were described using frequencies (percentage) for categorical variables, means and SDs for parametric continuous variables, and median and interquartile range (IQR) for skewed data.

## 3. Results

### 3.1. Clinical Characteristics and Manifestations

A total of 55 patients who were diagnosed with PB by bronchoscopic removal of tree-like casts were enrolled in the study. Demographic characteristics and the main clinical features of patients are shown in Table 1. The current study included 29 boys and 26 girls, with a male-to-female ratio of 1.12 : 1. The median age was 5.3 years (IQR: 3.0 to 7.8 years). There were 19 patients (34.5%) with underlying diseases, including asthma (*n* = 6), congenital heart disease (*n* = 3), bronchiolitis obliterans (*n* = 2), airway stenosis (*n* = 2), leukemia (*n* = 2), diffuse pan-bronchiolitis (*n* = 1), thalassemia after bone marrow transplantation (*n* = 1), hyper-IgE syndrome with nutcracker syndrome (*n* = 1), and nemaline myopathy (*n* = 1). All the patients had cough symptoms, of which 49 cases (89.1%) were accompanied by fever with a peak of (39.6 ± 0.8°C). The most common signs were pulmonary crackles (78.2%) and dyspnea (61.8%).

The results of routine laboratory tests and chest imaging are listed in Table 1. Except for 32 patients (58.2%) with elevated CRP, less than half of the patients had abnormal laboratory results. The most common chest imaging findings were patchy opacity (81.8%), followed by pulmonary consolidation (60.0%).

### 3.2. Pathogen Detection

**Figure 1:**
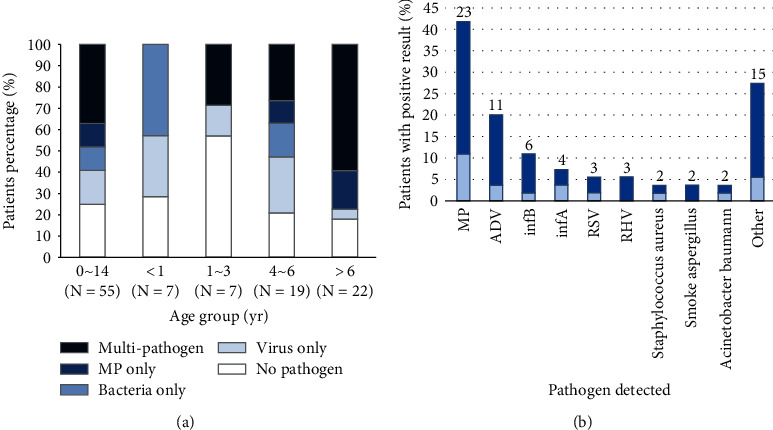
Pathogens detected in children with PB. (a) The proportion of pathogen types detected from January 2012 to May 2022, among 55 hospitalized children with bronchoscopic evidence of PB who had blood samples available for bacterial culture or real-time polymerase-chain-reaction (PCR) assays or endotracheal aspirate or BALF specimens available for bacterial culture and who also had nasopharyngeal or oropharyngeal swabs available for viral and atypical bacterial PCR assay or available viral serologic results. Panel b shows the numbers (above the bars) and percentages of all children in whom a specific pathogen was detected. Among 55 patients who had available tests for the detection of bacterial and viral pathogens, 41 were found to have a viral or bacterial pathogen (or both). Because more than 1 pathogen could be detected in a patient, a total of 15 pathogens other than those listed here were detected in 12 children, including cytomegalovirus (in 1), boca virus (in 1), enterovirus (in 1), other gram-positive bacteria (in 4), and other gram-negative bacteria (in 8). (b) Darker shading in the bar graph indicates that only the single pathogen was detected, and lighter shading indicates the pathogen was detected in combination with at least one other pathogen.

Among 41 patients who tested positive for respiratory pathogens, 24 cases (43.6%) had coinfections (≥2 pathogens tested positive). Pathogens were detected more commonly in children 4 years of age or older than in younger children (80.5% vs. 57.1%) (Figure 1(a)). The most detected pathogens were MP (in 41.8% of the children), adenovirus (ADV) (20.0%), influenza B virus (infB) (10.9%), influenza A virus (infA) (7.3%), respiratory syncytial virus (RSV) (5.5%), rhinoviruses (RHV) (5.5%), *Staphylococcus aureus* (3.6%), smoke *Aspergillus* (3.6%), and *Acinetobacter baumannii* (3.6%) (Figure 1(b)).

### 3.3. Bronchoscopic Manifestation and Intervention

**Figure 2:**
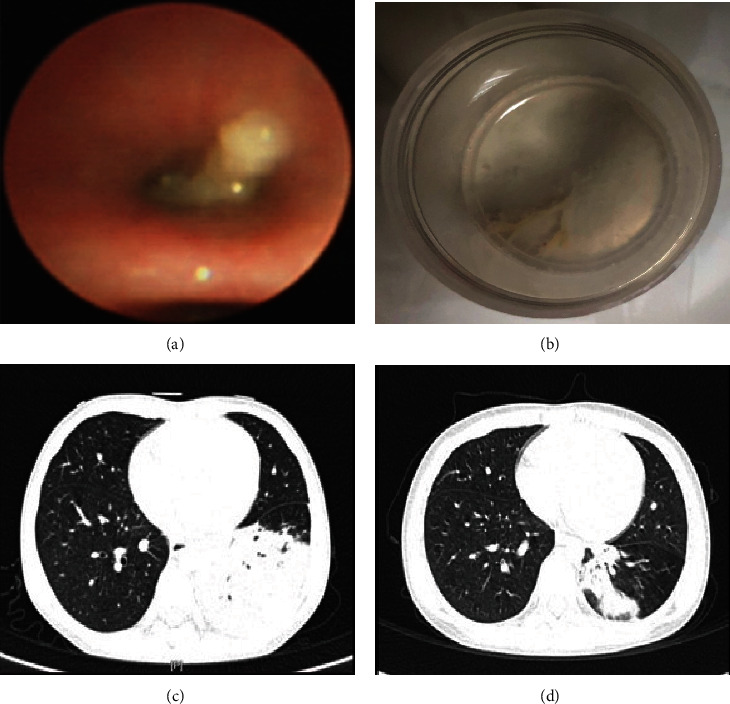
Bronchoscopic manifestation and chest CT in children with PB. (a) The bronchus in the left lower lobe blocked by a yellowish-white tough cast. (b) Tree-like cast removed by bronchoscopy. (c) Pulmonary consolidation in the lower left lung before treatment. (d) Absorption of lung consolidation after removal of cast.

All patients underwent bronchoscopy to remove the casts, and the median time of the first operation was the 3rd day after admission (IQR: 1-4.5 days). The times of bronchoscopies received by children with PB during hospitalization was 2.3 ± 1.9, including once in 20 cases (36.3%), twice in 17 (30.9%), thrice in 14 (25.5%), and more than three times in 4 (7.3%). Under the bronchoscope, the airway mucosa blocked by the casts was hyperemic and swollen, showing inflammatory stenosis (Figure 2(a)). Alveolar lavage via flexible bronchoscope was performed to all the patients, and a combination with other instruments was needed for some cases with hard sputum plug, including intrabronchial injection of ambroxol solution to resolve phlegm (*n* = 35, 63.6%) and utilization of biopsy forceps to clamp out the branching casts (*n* = 17, 30.9%). Cryoprobe extraction was performed on a patient whose mucus plug was jelly-like and fragile and could not be removed by suction or forceps. The suggested equipment and instructions are detailed in Box 1. The removed endophytic foreign body was commonly shaped like a bronchial tree or cord (Figure 2(b)), which was found in more than one site of the airway in 37 patients (67.3%). The blocking sites of the casts were as follows: left main bronchus (*n* = 4, 7.3%), left upper lobe (*n* = 23, 41.8%), left lower lobe (*n* = 30, 54.5%), right main bronchus (*n* = 5, 9.1%), right upper lobe (*n* = 11, 20%), right middle lobe (*n* = 21, 38.2%), right lower lobe (*n* = 20, 36.4%). After bronchoscopy, the children had improved ventilatory function and stronger respiratory sounds on the affected side. Postoperative chest imaging of 35 children showed that 29 cases (83%) were improved (Figures 2(c) and 2(d)). Eighteen cases underwent histopathological examination, 13 of which showed a large amount of fibrinous exudate with inflammatory cell infiltration, mainly neutrophils and eosinophils, which belonged to type I (inflammatory type) in the Seear standard classification [4].

### 3.4. Treatments and Outcome

All the patients received anti-infection, nebulization, and expectorant treatment, while some cases received invasive mechanical ventilation (*n* = 6, 10.9%), immunotherapy of gamma globulin (*n* = 25, 45.5%), and methylprednisolone treatment (*n* = 33, 60.0%). Fifty-three cases improved and were discharged with a median hospitalization time of 16 days (IQR: 11-24), while a total of 2 cases died, and the overall mortality rate was 3.6%. The two deceased patients had no underlying disease but were infected with multiple respiratory pathogens. One was a 12-year-old male who had infB combined with *Acinetobacter baumannii* infection and eventually died from severe pneumonia complicated by multiple organ failure. The other was a 4-year-old female with MP, ADV, infB, and *Staphylococcus epidermidis* infection, and eventually died from severe pneumonia and respiratory failure.

## 4. Discussion

PB is an uncommon and critical disease with a long history, and its specific incidence has not yet been clearly studied. Kunder et al. [3] collected the clinical data of 205,100 pediatric patients in the Stanford University medical institution over a 12-year period and concluded that the prevalence rate of PB in children was 6.8/100,000. However, due to the great differences in regional medical development levels, this data was probably because of referral patterns to a specialized center [3]. In the past, most of the studies on PB were reports of a few cases due to the rarity of the disease [5–7]. As the understanding of the disease deepened and bronchoscopy techniques developed, research on PB in children gradually increased.

Numerous systemic illnesses have been reported to be associated with PB, including Fontan cardiac surgery [8], asthma [9], nephrotic syndrome [10], and idiopathic chronic eosinophilic pneumonia [11]. About one-third of the children in our study had underlying disorders with scattered profiles, with asthma accounting for the largest proportion. A recent study found that a large number of inflammatory cells, mainly eosinophils and eosinophil extracellular traps (EETs), accumulated in the casts of PB associated with influenza virus infection, which indicated that the accumulation of eosinophils and their activation led to cytolytic extracellular trap cell death in the airway, culminating in mucus plugging [12]. Moreover, the study also showed that serum levels of IL-5, an essential cytokine for terminal differentiation of eosinophil precursors, were elevated in children with PB compared with those who did not develop PB [12]. Very little research has been done on the mechanisms of PB in children with asthma, and eosinophils may play an important role in this, which may well serve as a breakthrough for subsequent studies on the relationship between PB and allergic diseases.

In our study, more than 70% of the patients had a respiratory infection which is a common cause of PB, and the pathogens with the highest frequency of positive detection were MP, ADV, and infB, similar to other research results [13, 14]. Our study showed that patients of preschool and school age were the dominant group, and the respiratory infection rate of older children was significantly higher than that of infants (80.5% vs. 57.1%), which may be related to the age distribution of pathogens, as MP, the most detected pathogen, tends to infect older children.

Children with PB often have an acute onset, a short course of the disease, or an acute exacerbation. All the patients in our study had cough symptoms, and nearly 90% of them were accompanied by fever, mainly high fever, while lung moist rales and dyspnea are the most common signs we found, which was consistent with other studies [13, 15]. Laboratory examination results of PB patients are usually nonspecific, and the most common abnormal results in our study were elevated white blood cell count and CRP and coagulation dysfunction, suggesting that the onset of PB was likely to be accompanied by a systemic inflammatory reaction and may also be caused by infection. Some studies have found that the comprehensive evaluation of clinical indicators such as fever peak, neutrophil ratio, platelet count, IL-6, and LDH was helpful for predicting whether severe *Mycoplasma pneumoniae pneumonia* (MPP) would complicate PB [16, 17], which still needs prospective studies with larger sample sizes to assess the sensitivity.

Compared to nonspecific abnormal laboratory test results, chest imaging is considered more valuable for PB diagnosis and evaluation for interventional therapy. Patchy exudates and consolidation were the most common chest CT findings due to partial airway obstruction caused by the casts. Pleural effusion was also common that occurred in nearly half of the children in this study, especially in patients with MP infection, which was in line with the imaging features of MPP [18]. Therefore, once the patient develops a high fever, cough, and dyspnea within a short time, and the chest imaging shows atelectasis, especially if the pathogens closely related to PB such as MP and some respiratory viruses are detected positive, the possibility of PB should be considered. At this point, patients should be referred for subspecialty assessment and prompt implementation of active treatment measures.

Bronchoscopy intervention is an important means of treating PB, and removal of the cast as soon as possible through bronchoscopy is the most effective way to relieve airway obstruction. The median time for the first bronchoscopy performed in children with PB in our study was the third day after admission, and most children required multiple bronchoscopies. Sputum plugging was seen more frequently in the left lung under bronchoscopy, most likely related to the narrow diameter of the left main bronchus and its greater angle from the midline, which makes secretions difficult to extract. Casts in the left lung were more common in the lower lobe and those in the right lung were more common in the middle lobe, which was thought to be related to poor drainage of secretions due to their anatomical location. All the patients underwent alveolar lavage, but most of the casts were relatively hard and firmly set, and simple sucking was sometimes difficult to achieve satisfactory effects, so we prefer to infuse ambroxol into the airway during the operation to soften the mold, while some studies found that using other mucolytics such as topical tissue-type plasminogen activator and rhDNase was also effective [19, 20]. About one-third of the children in the study needed forceps to remove the casts, and regular biopsy forceps with a diameter of 1.8 mm was used for the casts in the proximal airway and 1.2 mm for the small distal airway. For some jelly-like casts with high moisture content that could not be completely cleaned by alveolar lavage and were easy to be broken by forceps, we tried to use a freezing probe to freeze them for 3~5 s and then pull them out of the airway, and the casts could be removed successfully. There have been only a few reports of cryosurgery applications in children with PB, and more cases are needed to assess its indications and therapeutic value. Operating bronchoscopy in children with PB is fraught with challenges, as in addition to the limited operating space caused by the small airways of the child, the patients also suffer from airway obstruction and restricted ventilation due to the casts. The procedure of bronchoscopy is therefore extremely demanding and should be performed in as short a time as possible, while attempting to remove the cast, so as not to aggravate the hypoxia of the patient. In this study, bronchoscopies were performed by clinicians with extensive experience and completed successfully without serious complications. Patients experienced rapid improvement in ventilation after removal of the casts, and reexamining chest imaging showed improvement in more than 80 percent of the children.

## 5. Conclusions

In summary, PB in children is a rare and serious life-threatening respiratory disease with acute onset of airway obstruction as the most typical clinical feature, and removal of the cast by bronchoscopy is the most effective treatment. We summarize the clinical characteristics and experiences of bronchoscopy in children with PB admitted to our hospital over the last 10 years to improve clinicians' understanding of the disease. However, this study was only a single-center retrospective study, and the number of patient samples and information collected was limited. Follow-up multicenter prospective studies should be conducted to better explore the pathogenesis of PB and discover factors that affect the disease prognosis.

## Figures and Tables

**Box 1:**
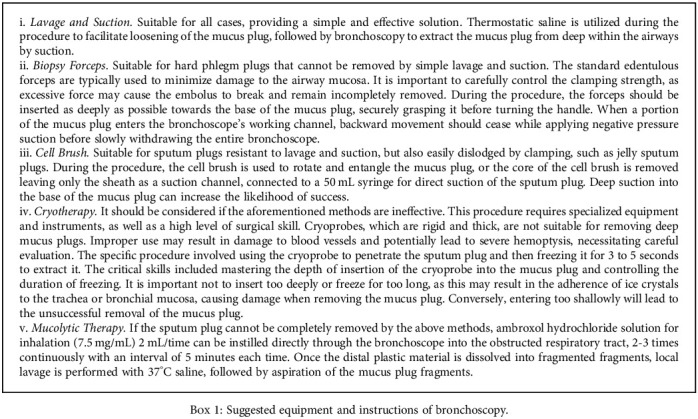
Suggested equipment and instructions of bronchoscopy.

**Table 1:** Characteristics of children with plastic bronchitis.

Characteristics	Children with Bronchoscopic evidence of plastic bronchitis (*n* = 55)
Age group—no. (%)	
<1 yr	7 (12.7)
1~3 yr	7 (12.7)
4~6 yr	19 (34.6)
>6 yr	22 (40.0)
Any underlying condition—no. (%)	19 (34.5)
Asthma	6
Congenital heart disease	3
Others	10
Course of the disease—days before admission	
Median	11
Interquartile range	6-30
Symptom—no. (%)	
Cough	55 (100)
Fever	49 (89.1)
Wheeze	23 (41.8)
Sign—no. (%)	
Moist rales	43 (78.2)
Dyspnea†	34 (61.8)
Diminished respiration	30 (54.5)
Wheezing sounds	19 (34.5)
Laboratory findings—no. (%)	
PaO_2_/FiO_2_<200 – no./total no. (%)	11/40 (27.5)
PaCO_2_>45 mmHg – no./total no. (%)	6/40 (15.0)
Blood leukocyte count	
>10.0 × 10^9^/L	22 (40.0)
<4.0 × 10^9^/L	7 (12.7)
CRP level >0.6 mg/dL	32 (58.2)
PT>14.5 s/APTT >42.8 s	26 (47.3)
CK-MB >25 U/L	15 (27.3)
ALT >40 U/L	14 (25.5)
Chest imaging findings—no. (%)	
Patchy opacity	45 (81.2)
Consolidation	33 (60.0)
Pleural effusion	24 (43.6)
Air leak‡	7 (12.3)

†Signs of dyspnea included shortness of breath, nares flaring, and three concave signs. ‡Chest imaging findings of air leak included pneumothorax, mediastinal emphysema, and subcutaneous emphysema. PT: prothrombin time; APTT: activated partial thromboplastin time; CK-MB: creatine kinase-MB; ALT: alanine aminotransferase.

## Data Availability

The data that support the findings of this study are available on request from the corresponding author. The data are not publicly available due to privacy or ethical restrictions.
